# Disparities in Incidences of Cesarean Section Among Women With Gestational Diabetes Mellitus in the United States

**DOI:** 10.7759/cureus.29400

**Published:** 2022-09-21

**Authors:** Oluwasegun A Akinyemi, Christina Lipscombe, Ofure V Omokhodion, Akinwale S Akingbule, Mojisola E Fasokun, Oluwagbemiga A Oyeleye, Resham Tanna, Bolarinwa Akinwumi, Kindha Elleissy Nasef, Mary Fakorede

**Affiliations:** 1 Department of Health Policy and Management, University of Maryland School of Public Health, College Park, USA; 2 Surgery, Howard University, Washington, DC, USA; 3 Department of Obstetrics and Gynecology, Howard University College of Medicine, Washington, DC, USA; 4 Department of Obstetrics and Gynecology, University College Hospital, Ibadan, NGA; 5 General Practice, Primary Care, Public Health, Ozark Valley Medical Clinic, Branson, USA; 6 Department of Epidemiology and Public Health, University of Alabama at Birmingham, Birmingham, USA; 7 Department of Internal Medicine, Lagos University Teaching Hospital, Lagos, NGA; 8 Department of Obstetrics and Gynecology, Spartan Health Sciences University, Vieux Fort, LCA; 9 Department of Health Sciences and Social Work, Western Illinois University, Macomb, USA; 10 Department of Family Medicine, Howard University College of Medicine, Washington, DC, USA; 11 Psychiatry, Ladoke Akintola University, Ogbomoso, NGA

**Keywords:** race/ethnicity, insurance type, cesarean section, gestational diabetes mellitus, disparity

## Abstract

Background

In this study, we explored the interaction between women’s race/ethnicity and insurance type and determined how these interactions affect the incidences of cesarean section (CS) among women with gestational diabetes mellitus (GDM).

Methodology

We utilized the National Inpatient Sample (NIS) database from January 2000 to September 2015 to conduct a retrospective analysis of all GDM-associated hospitalizations. We then explored the interaction between race/ethnicity and insurance types and determined how these interactions affect the incidences of CS among GDM patients, controlling for traditional risk factors for CS and patients’ sociodemographics. Subsequently, we determined the risk of primary postpartum hemorrhage (PPH) in the CS group and a propensity score-matched control group who had vaginal deliveries.

Results

There were 932,431 deliveries diagnosed with GDM in the NIS database from January 2000 to September 2015. The mean age of the study population was 30.6 ± 5.9 years, 44.5% were white, 14.0% were black, and 26.7% were Hispanic. The CS rate was 40.5%. After controlling for covariates, women who utilized private insurance had the highest CS rate across the different races/ethnicities; white (odds ratio (OR) = 1.21 (1.17-1.25)) blacks (OR = 1.33 (1.26-1.41)), and Hispanic (OR = 1.12 (1.06-1.18)). CS patients were less likely to develop PPH compared to their matched controls with vaginal deliveries (OR = 0.67 (0.63-0.71)).

Conclusions

Private insurance is associated with higher incidences of CS among women with GDM, irrespective of race/ethnicity.

## Introduction

Gestational diabetes mellitus (GDM) is the occurrence of glucose intolerance with the onset or first recognition during pregnancy [1]. The prevalence of GDM worldwide is about 7-10% of all pregnancies [2]. In the United States, 2-10% of all pregnancies develop GDM annually. Screening for GDM usually occurs after 24 weeks in asymptomatic women or those without risk factors for diabetes mellitus [3]. However, women with risk factors for GDM should undergo screening during the first antenatal care visit.

Risk factors for GDM include a previous history of GDM, pre-pregnancy obesity, advanced maternal age, diabetes in a first-degree relative, and ethnic minorities such as blacks, Native Americans, Pacific Islanders, or Hispanics. Between 2006 and 2016, the prevalence of GDM increased by 3.6%. The increase was more prominent among the overweight, non-white population and those with a low income. The American Diabetes Association recommends the one or two-step approach to screening GDM [4]. In the one-step approach, women receive a 75 g oral glucose load followed by fasting, one, and two-hour glucose measurements. In the two-step approach, women initially receive a 50 g glucose load followed by a 100 g glucose challenge.

Women with GDM have a higher incidence of delivery by cesarean section (CS) [5]. This is often a result of a higher incidence of fetal macrosomia and other comorbidities. Though increased CS has been associated with higher adverse outcomes among women, this may not be entirely true among women with GDM [6]. CS may lead to reduced shoulder dystocia, perineal laceration, and primary postpartum hemorrhage (PPH) in carefully selected women. In addition, CS may lead to improved pregnancy outcomes among women with suspected fetal macrosomia and other complications.

In the United States, social determinants of health such as access to insurance, median income, and race/ethnicity influence access to many lifesaving procedures [7]. Several studies have highlighted these disparities in the incidences of CS [8]. While studies have highlighted a higher incidence of primary CS among minority groups, there is a need to further explore the interaction of patients’ race/ethnicity and insurance types and how these affect the incidences of CS.

In the present study, we explored the interaction between race/ethnicity and insurance types and how these interactions influence the incidences of CS among women with GDM.

## Materials and methods

Data sources

This study is a retrospective analysis of all hospitalizations with the diagnosis of GDM included in the National Inpatient Sample (NIS) database (2000-2015). The NIS is maintained as part of the Health Care Utilization Project (HCUP) of the Agency for Healthcare Research and Quality (AHRQ). It is the largest all-payer inpatient database in the United States and comprises a 20% stratified random sample of all US hospital discharges. Each of these discharges in the NIS is de-identified. Further details on the NIS design are available at: https://www.hcup-us.ahrq.gov/.

Study population

There were 932,431 GDM-associated hospitalizations in the NIS from January 2000 to September 2015. We used the current International Classification of Diseases, Ninth Revision diagnosis code 648.8x to define GDM. We excluded all hospitalizations in the fourth quarter of 2015, ages <18 or >49 years, and those with missing variables. The exclusion of all hospitalizations in the fourth quarter of 2015 accounted for the transition from the ICD-9 to ICD-10 codes on October 1, 2015.

Patient characteristics and risk factors

We stratified patients’ race/ethnicity as white, black, Hispanic, Asian/Pacific Islanders, Native Americans, and others, and insurance types as private, public, and uninsured. Then, utilizing the HCUP dataset variable zip, we grouped the annual median income into four quartiles, with quartile I corresponding to the lowest income quartile and quartile IV corresponding to the highest income quartile.

Definition of study outcomes

Our primary outcome was delivery by CS. We identified the current ICD-9 diagnosis codes 740, 741, 742, 744, and 7499 for CS. Additional analyses included a propensity score-matched analysis to compare the incidences of PPH and length of hospital stay among women who underwent CS and a matched control who had a vaginal delivery.

Propensity score matching

Propensity score matching is a method to measure the difference in outcomes between groups (usually a target group and a control group) when randomization is impossible. A significant limitation of observational studies is the lack of randomization. It is impossible to infer the outcomes between two groups (a target group and a control group). Patients with specific characteristics may be more likely to develop the study outcome. We employed propensity score matching in this study to eliminate the impact of variables such as maternal age, day of admission, previous CS, malpositioning, cephalopelvic disproportion (CPD), hypertension, pre-pregnancy obesity, race/ethnicities, and annual median income on the incidences of PPH between the two groups.

Statistical analysis

We utilized frequencies and percentages to describe the baseline characteristics of patients and outcome variables, and Pearson chi-square tests to evaluate the relationship between studied variables and the incidences of CS. We utilized multivariate logistic regression with interaction term analysis to determine the association between race/ethnicity, insurance types, and incidences of CS. Estimates were expressed as adjusted odds ratios (ORs) and 95% confidence intervals (CIs). In a subanalysis, we utilized a nearest neighbor propensity score matching without replacement in a 1:1 ratio, with a caliper width set to 0.02 times the standard deviation (SD) of the propensity score to compare the incidence of PPH and length of hospital stay between women who underwent CS and a control group who delivered via vaginal delivery.

A two-tailed p-value of <0.05 was considered statistically significant. All statistical analyses were performed using STATA 14 (StataCorp., College Station, TX, USA).

## Results

From January 2000 to September 2015, there were 932,431 hospitalizations in the United States with a primary diagnosis of GDM. This is about 3.4% of the total hospitalizations during this period and lies within the Centers for Disease Control and Prevention (CDC) range of 2-10% prevalence. Table 1 shows the demographic distribution of these patients by race/ethnicity.

**Table 1:** Baseline characteristics by race/ethnicity (2000-2015) CS: cesarean section; CPD: cephalopelvic disproportion

Variable	Whites (n = 344,082)	Blacks (n = 108,512)	Hispanics (n = 206,236)	Native Americans (n = 72,544)	Other (n = 41,899)	P-value
Age						<0.001
<35 years	73.12%	74.93%	71.45%	64.66%	70.71%	
≥35 years	26.88%	25.07%	28.55%	35.34%	29.29%	
Median income						<0.001
First quartile (	18.83%	41.30%	34.34%	12.70%	22.37%	
Second quartile ($42,000–$51,999)	24.94%	23.53%	25.58%	15.66%	22.43%	
Third quartile ($52,000–$67,999)	27.12%	19.61%	23.02%	22.65%	25.38%	
Fourth quartile (>$68,000)	29.11%	15.56%	17.06%	48.99%	29.82%	
Insurance						<0.001
Private	65.36%	38.28%	30.36%	68.22%	46.58%	
Public	30.21%	18.64%	62.07%	26.56%	44.08%	
Uninsured	4.43%	4.74%	7.57%	5.22%	9.34%	
Previous CS	20.53%	21.94%	24.59%	19.37%	20.86%	<0.001
Hypertension	20.37%	31.19%	16.63%	12.91%	15.27%	<0.001
CPD	5.72%	4.75%	5.03%	5.40%	5.28%	<0.001
Prolonged pregnancy	3.71%	2.64%	4.25%	5.40%	4.64%	<0.001
Obesity	11.16%	17.95%	10.65%	5.46%	7.41%	<0.001
Malpresentation	9.27%	7.04%	7.89%	9.05%	8.67%	<0.001
CS	42.29%	40.90%	40.02%	38.41%	39.87%	<0.001

Table 2 is a bivariate analysis showing the association between various independent variables and the risk of CS. The table shows the significant relationship between traditional risk factors for CS, social determinants of health variables such as race, median income, and insurance type, and delivery by CS.

**Table 2:** Bivariate analysis showing the association between studied variables and delivery types (2000-2015). CS: cesarean section; CPD: cephalopelvic disproportion; LOS: length of hospital stay; PPH: primary postpartum hemorrhage

Variables	Total population	Cesarean section		P-value
	(N = 932,431)	Yes (n = 377,720)	No (n = 554,539)	
Age	30.65 ± 5.92	31.33 ± 5.87	30.17 ± 5.91	<0.001
Race/Ethnicity				<0.001
Whites	344,082	42.29%	57.71%	
Blacks	108,512	40.90%	59.10%	
Hispanics	206,236	40.02%	59.98%	
Native Americans	72,544	38.41%	61.59%	
Others	41,899	39.87%	60.13%	
Median income				<0.001
First quartile (	225,865	40.52%	59.48%	
Second quartile ($42,000–$51,999)	223,761	40.18%	59.82%	
Third quartile ($52,000–$67,999)	226,443	40.71%	59.29%	
Fourth quartile (>$68,000)	238,874	40.67%	59.33%	
Insurance				<0.001
Private	487,955	42.45%	57.55%	
Public	390,744	38.82%	61.18%	
Uninsured	52,215	35.17%	64.83%	
Previous CS	198,496	86.09%	13.91%	<0.001
Hypertension	184,081	50.38%	49.62%	<0.001
CPD	51,526	59.34%	40.66%	<0.001
Prolonged pregnancy	35,622	34.05%	65.95%	<0.001
Obesity	76,619	55.18%	44.82%	<0.001
Malpresentation	79,848	84.72%	15.28%	<0.001
LOS	3.32 ± 4.25	4.08 ± 4.71	2.80 ± 3.82	<0.001
Primary PPH	20,342	35.35%	64.65%	<0.001

**Figure 1:**
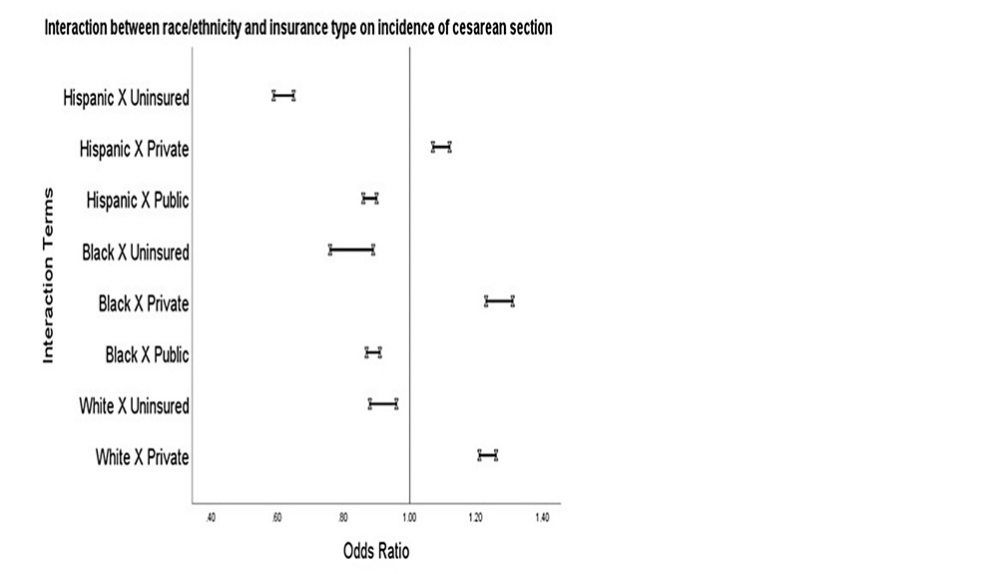
Interaction between race/ethnicity and insurance types and association with incidence of cesarean section (2000-2015). x: interaction term analysis

Table 3 and Figure 1 show the interaction between race, insurance type, and access to CS control for traditional CS risk factors. Across all races/ethnicities, women who utilized private insurance had the highest CS rates.

**Table 3:** Interaction between race/ethnicity, insurance type, and access to CS among GDM patients in the United States (2005-2015). † Model adjusted for age, premorbid hypertension, previous CS, income, CPD, prolonged pregnancy, pre-pregnancy obesity, and malpositioning. CS: cesarean section; GDM: gestational diabetes mellitus; CPD: cephalopelvic disproportion

Interaction terms	Odds ratio	95% confidence interval		P-value
White X Public insurance	Reference	Lower CI	Upper CI	
White X Private	1.21	1.17	1.25	<0.001
White X Uninsured	0.86	0.8	0.93	<0.001
Black X Private	1.33	1.26	1.41	<0.001
Black X Public	0.9	0.86	0.95	<0.001
Black X Uninsured	0.87	0.75	1.02	0.08
Hispanic X Private	1.12	1.06	1.18	<0.001
Hispanic X Public	0.81	0.77	0.85	<0.001
Hispanic X Uninsured	0.6	0.54	0.67	<0.001
Asian/Pacific/Native American X Private	1.05	0.99	1.1	0.1
Asian/Pacific/Native American X Public	0.84	0.78	0.91	<0.001
Asian/Pacific/Native American X Uninsured	1.06	0.92	1.22	0.44

Table 4 is a propensity score-matched model comparing the risk of PPH and length of hospital stay among women who had CS and matched controls whose mode of delivery was a vaginal delivery. There was a 40% increase in PPH among the matched controls.

**Table 4:** Selected outcomes by mode of delivery after propensity score matching (2000-2015). † Model matched for age, premorbid hypertension, previous CS, income, CPD, prolonged pregnancy, pre-pregnancy obesity, malpositioning, race/ethnicity, and annual income. LOS: length of hospital stay; PPH: post-partum hemorrhage; CS: cesarean section; CPD: cephalopelvic disproportion

Variables	Total population (N = 755,308)	Cesarean section (n = 377,654)	Vaginal delivery (n = 377,654)	P-value
Primary PPH	16,640	43.21%	56.79%	<0.001
LOS	755,308	4.08 ± 4.71	2.87 ± 4.00	<0.001

## Discussion

In this study, we explored the interaction between race/ethnicity and insurance type and determined how these interactions affect the incidence of CS among women with GDM. We found that GDM accounted for 3.4% of hospitalizations between 2000 and 2015. Of these women, the majority were whites, followed by Hispanics and blacks. This contrasts with Shah et al. [9], who found the highest rates of GDM among Hispanics. They found a GDM prevalence of 6%, and the difference from our study findings may be due to the over-representation of Hispanics in their study population.

In this study, the CS rate was higher among GDM patients than that of the general population in 2020 (40.5% versus 31.8%, respectively), as reported by the CDC [10]. This may represent an independent association between GDM and CS rate due to the higher incidence of fetal macrosomia and other indications for cesarean delivery, including obesity and previous cesarean delivery.

Though we found a higher prevalence of previous CS among black women with GDM, they had a lower incidence of CS in the index delivery compared to white women. Other studies have found mixed results, with some demonstrating no statistically significant difference in the incidences of CS among the different races/ethnicities [11], while others showed a higher incidence among black women compared to other races/ethnicities [12].

Women with private insurance have a higher CS rate than those with public or uninsured insurance. Across all races/ethnicities, patients with private insurance have a higher CS rate. Our finding aligns with other reports in the literature. In a systematic review and meta-analysis by Hoxha et al., patients with private insurance had the highest CS rates across all 18 studies [13]. Uninsured women had the lowest CS rates across all races/ethnicities. These findings highlight the influence of socioeconomic status on CS rates in the United States and may imply that financial incentives may influence the decisions for cesarean delivery.

Further, black women with private insurance had the highest rates of cesarean delivery despite white women having the highest proportions of private insurance coverage. Our initial finding of the highest CS rates among white women was before controlling for other maternal factors. This new finding of the highest rates among black women with private insurance may reflect an independent association between ethnicity and cesarean delivery among women with GDM. Thus, racial or ethnic factors may predispose women to have a cesarean delivery. This needs further exploration.

Interestingly, women who had cesarean delivery had a lower incidence of PPH than their counterparts who had a vaginal delivery. This may be because the threshold for PPH is higher for cesarean delivery (1000 mL). The difference may also reflect that blood loss from cesarean delivery is often under-reported. It has also been noted that transfusions after CS for low hemoglobin are usually not considered in reporting [14]. Despite this finding, several studies have found a higher incidence of adverse pregnancy outcomes across maternal and neonatal outcomes among patients who underwent CS compared to those who had vaginal deliveries [15,16].

A limitation of this study is the use of administrative databases, as these databases are primarily for reimbursement purposes and are prone to coding errors. However, this study addresses a gap in knowledge about the independent association between insurance coverage and CS rates in women with GDM. The robust nature of the dataset also lends credibility to the results.

## Conclusions

Private insurance is associated with higher CS rates in women with GDM, irrespective of ethnicity, and CS rates among women with GDM are alarmingly high. Therefore, financial incentives from private insurance may play a role in the rising CS rates, and policies addressing insurance protocol and reimbursements may be the key to reducing CS rates in this population.
